# Spontaneous cutaneous adverse drug reaction reports—An analysis of a 10‐year dataset in Singapore

**DOI:** 10.1002/prp2.469

**Published:** 2019-03-13

**Authors:** Si Xian Wong, Mun Yee Tham, Chee Leok Goh, Han Hui Cheong, Sui Yung Chan

**Affiliations:** ^1^ Department of Pharmacy Faculty of Science National University of Singapore Singapore Singapore; ^2^ Vigilance and Compliance Branch Health Sciences Authority Singapore Singapore; ^3^ National Skin Centre Singapore Singapore; ^4^ Department of Pharmacy KK Women's and Children's Hospital Singapore Singapore

**Keywords:** adverse drug reactions pharmacovigilance, skin, spontaneous reporting

## Abstract

We analyzed the spontaneous adverse event database in Singapore to determine the types of cutaneous adverse drug reactions (CADRs) and causative drugs reported. We selected 10 CADRs‐of‐interest, and identified the suspected drugs and the characteristics of the at‐risk population. ADR reports received from 2006 to 2015 of the system organ class “Skin and Appendages Disorders” were analyzed based on patient demographics, the types of CADRs, suspected drugs, outcome, and latency period. Of the 104 372 reports analyzed, 56.2% involved females and 72.5% involved Chinese patients. The mean age was 41.1 years old. The top CADRs reported were rash (including nonspecified rash, follicular rash, maculopapular rash, and vesicular rash) (67.2%) and angioedema (13.9%). The drugs frequently associated with the CADRs‐of‐interest include nonsteroidal antiinflammatory drugs and antibiotics with angioedema, iohexol with urticaria, and antiepileptics and allopurinol with Stevens‐Johnson syndrome (SJS)/toxic epidermal necrolysis (TEN). A subgroup analysis based on age, sex, and race on the 10 CADRs‐of‐interest showed the following trends in reporting: Alopecia (reported more in females), drug hypersensitivity syndrome (more in males), angioedema (more in younger patients), and photosensitivity (more in older patients). In general, the racial distribution across each CADR‐of‐interest was consistent with that of Singapore's population, with slight deviations observed for SJS/TEN, photosensitivity and skin discoloration. We analyzed CADR reports from Singapore over 10 years, and identified the types of CADRs reported, and their associated drugs, latency periods and patient characteristics. Such information could add value to healthcare professionals as they assess CADR cases and evaluate suspected drugs.

**Abbreviations**
ADR: adverse drug reaction
CADRs: cutaneous ADRs
CADRs: cutaneous adverse drug reactions
SJS: Stevens‐Johnson syndrome
SOC: system organ class
TEN: toxic epidermal necrolysis
WHO: World Health Organisation

## 1 INTRODUCTION

According to the World Health Organisation (WHO), an adverse drug reaction (ADR) is a response to a medicinal product which is noxious and unintended and which occurs at doses normally used in man.^1^ Cutaneous ADRs (CADRs) are one of the most common ADRs,^2^, ^3^, ^4^ with an overall incidence rate of 2%‐3% in hospitalized patients.^5^ In the WHO global ADR database, VigiBase, skin and appendages disorders account for 18.3% of over 13 million ADR reports received from more than 100 countries, making it the third most frequently reported system organ class (SOC).^6^, ^7^ As the national regulatory agency in Singapore, the Health Sciences Authority (HSA) receives around 20 000 ADR reports annually in the adverse event (AE) database, of which 60% were related to skin reactions.

The manifestation of CADRs can be very varied, ranging from mild, self‐limiting reactions to severe cutaneous adverse reactions (SCARs) associated with significant morbidity and mortality, such as acute generalized exanthematous pustulosis (AGEP), Stevens‐Johnson syndrome (SJS), toxic epidermal necrolysis (TEN), and drug hypersensitivity syndrome (DHS). CADRs are also associated with a wide range of drugs, with antimicrobials, NonSteroidal Anti‐Inflammatory Drugs (NSAIDs), antiepileptics, and analgesics as the most frequently implicated drug classes.^8^, ^9^, ^10^, ^11^, ^12^ While much is known about CADRs, information on the types of CADRs reported through the spontaneous ADR reporting system is limited. We seek to fill this knowledge gap with an analysis of a large dataset with over 100 000 CADR reports from a 10‐year period from the HSA AE database. Our objectives are to determine the types of CADRs and associated drugs reported to the HSA AE database, to identify characteristics of the at‐risk population, and to identify associations between CADRs and drugs.

## 2 MATERIALS AND METHODS

### 2.1 Data source

In Singapore, spontaneous AE reports are submitted to HSA and captured into the national AE database. These reports come primarily from healthcare professionals via the Critical Medical Information Store (CMIS) or by email, online, fax, or post. The CMIS, a data repository for ADRs, drug allergies and medical alerts, allows healthcare professionals to enter AE information into the patient's electronic medical record, and this information is then transmitted to HSA, making reporting of AEs a seamless process. Since the introduction of CMIS in 2006, the number of reports received by HSA has increased exponentially, from 1185 reports in 2005 to 10 685 in 2006 and stabilizing at about 20 000 reports annually since 2010, facilitating the detection of potential drug safety signals.

For each report, AEs were coded using the WHO Adverse Reaction Terminology (WHO‐ART), drugs were classified using the Anatomical Therapeutic Chemical (ATC) Classification System, and causality was assessed based on the WHO Uppsala Monitoring Centre (WHO‐UMC) causality assessment system.

### 2.2 Inclusion criteria

Spontaneous AE reports which met the following criteria were included in our study: (1) report was received between 2006 and 2015, (2) reported AE belongs to the SOC of “Skin and Appendages Disorders” and (3) causality was assessed as certain, probable or possible.

### 2.3 Data extraction, collation, and analysis

Anonymized data with basic demographic information, reporters’ profession, AE description, patient outcome, and suspected drug(s) were extracted from the AE database.

To facilitate analysis, WHO‐ART preferred terms used for coding the CADRs were grouped into 21 reaction types. From these, 10 CADRs‐of‐interest were selected for in‐depth analysis based on clinical relevance.

### 2.4 Statistical analysis

All variables were analyzed by applying descriptive statistics using Statistical Package for the Social Sciences (SPSS) Inc., Chicago, IL, USA, version 24.0.

This study was approved by the National University of Singapore Institutional Review Board.

## 3 RESULTS

Out of 178 810 AE reports captured between 2006 and 2015, 104 372 AE reports belonging to the SOC of “Skin and Appendages Disorder” were included in our analysis, and their characteristics are provided in Table 1. The mean patient age was 41.1 years old, with a bimodal distribution peaking at 20‐29 years old (16.9%) and 50‐59 years old (15.7%). A majority of the CADR reports involved Chinese (72.5%), in tandem with the country's demographics, and female patients (56.2%). Most of the CADRs were reported by doctors (91.9%), and were associated with Western health products (98.6%). About slightly more than half of the CADRs reported were assessed as nonserious (59.2%).

**Table 1:** Demographic characteristics of patients and characteristics of reports with AEs belonging to SOC “Skin and Appendages Disorder”

Demographics of patients	%	Characteristics of report	%
Sex^a^		Types of Product	
Male	43.8	Western	98.6
Female	56.2	Vaccines	0.5
Age (Mean = 41.1 years old;		Health supplements	0.4
Range = 0 to 115 years old)^a^		Biologics (excluding vaccines)	0.3
0‐9	6.6	Complementary medicine	0.1
10‐19	10.5	Cosmetics	<0.1
20‐29	16.9	Medical device	<0.1
30‐39	14.2	Others	<0.1
40‐49	14.4	Information unavailable	<0.1
50‐59	15.7	Assessment of causality	
60‐69	11.4	Possible	97.1
70‐79	6.7	Probable	2.4
≥ 80	3.5	Certain	0.5
Race^a^		Assessment of seriousness^b^	
Chinese	72.5	Serious	40.8
Malay	12.5	Not Serious	59.2
Indian	8.1	Reporter's profession	
Others	7.0	Doctor	91.9
		Pharmacist	6.4
		Dentist	0.7
		Nurse	0.4
		Drug company	0.4
		Research coordinator	< 0.1
		Others	< 0.1
		Consumer	< 0.1

a Out of 104 372 CADR reports, sex, age, and race were not reported in 2.8%, 5.9%, and 12.1% of the reports, respectively, and these were excluded from the figures.

b Seriousness is assessed based on the International Conference of Harmonisation (ICH) E2A guidelines.

The types of CADRs reported and their frequencies are listed in Table 2. Rash (including nonspecified rash, follicular rash, maculopapular rash, and vesicular rash) was most frequently reported (67.2%), followed by angioedema (13.9%) and pruritus (7.4%). SCARs such as SJS/TEN, AGEP and pustular rash, and DHS were reported less frequently, in 0.7%, 0.5%, and 0.2% of the reports, respectively.

**Table 2:** Types of CADRs reported and their frequencies

No.	Reaction type	Frequency (N)	%
‐	Total	108 798^a^	100.00
1	Rash (includes nonspecified rash, follicular rash, maculopapular rash, vesicular rash)	73 074	67.2
2	Angioedema	15 177	13.9
3	Pruritus	8065	7.4
4	Urticaria	7704	7.1
5	Stevens‐Johnson Syndrome and Toxic Epidermal Necrolysis (SJS/TEN)	813	0.7
6	Fixed Drug Eruption (FDE)	790	0.7
7	Acute Generalized Exanthematous Pustulosis (AGEP) and pustular rash	510	0.5
8	Bullous eruption	488	0.4
9	Erythema multiforme	311	0.3
10	Generalized exfoliative dermatitis	296	0.3
11	Dermatitis (includes eczema, contact dermatitis, nonspecified dermatitis, dermatitis lichenoid, seborrheic dermatitis)	261	0.2
12	Drug Hypersensitivity Syndrome (DHS)	252	0.2
13	Sweat Gland Disorder (includes decreased sweating, increased sweating, and sweat gland disorder)	193	0.2
14	Purpura (includes purpuric rash)	180	0.2
15	Skin exfoliation	160	0.1
16	Photosensitivity (includes photoallergic reaction, nonspecified photosensitivity, phototoxic reaction)	113	0.1
17	Alopecia	69	0.1
18	Psoriasiform eruptions (includes psoriaform rash, psoriasis)	66	0.1
19	Skin discoloration (includes skin depigmentation, vitiligo, nonspecified skin discoloration, chloasma, pigmentation abnormal)	56	0.1
20	Acneiform eruptions	29	< 0.1
21	Others	193	0.2

a Total number of CADR report included in analysis = 104 372; Number of CADRs frequency > 104 372 as there could be more than one type of CADR reported in a single report.

Table 3 shows the top 10 drug classes, and the respective top five drugs in each class. Systemic antibacterials were most commonly implicated (43.5%) followed by antiinflammatory and antirheumatic products (16.2%) and analgesics (9.0%). Antiepileptics made up 1.6% of the suspected drugs, and was ranked seventh.

**Table 3:** Top 10 drug classes reported with CADRs, and top five suspected drugs per drug class

No.	Drug classes (ATC Code – Level 2) and suspected drugs	No. of reports	%
1	Antibacterials for systemic use – J01	48 874	43.5
	Amoxicillin	8511	
	Cotrimoxazole	6921	
	Coamoxiclav	6609	
	Benzylpenicillin or Penicillin G	2787	
	Erythromycin	2714	
2	Antiinflammatory and Antirheumatic products – M01	18 265	16.2
	Ibuprofen	4185	
	Diclofenac	4006	
	Naproxen	3234	
	Mefenamic Acid	2922	
	Etoricoxib	1214	
3	Analgesics – N02	10 112	9.0
	Paracetamol	6871	
	Tramadol	1205	
	Orphenadrine, Paracetamol	742	
	Codeine	566	
	Morphine	202	
4	Antithrombotic Agents – B01	4353	3.9
	Aspirin	3915	
	Clopidogrel	244	
	Ticlopidine	115	
	Dipyridamole	47	
	Warfarin	32	
5	Lipid Modifying Agents – C10	2566	2.3
	Simvastatin	1188	
	Fenofibrate	472	
	Lovastatin	322	
	Atorvastatin	239	
	Laropiprant and Niacin	100	
6	Contrast Media – V08	1922	1.7
	Iohexol	1500	
	Ioversol	135	
	Gadoterate	65	
	Iopamidol	65	
	Iopromide	65	
7	Antiepileptics – N03	1865	1.6
	Phenytoin	697	
	Carbamazepine	482	
	Gabapentin	190	
	Lamotrigine	174	
	Valproate	158	
8	Drugs for Acid Related Disorders – A02	1578	1.4
	Omeprazole	982	
	Famotidine	234	
	Esomeprazole	95	
	Ranitidine	92	
	Magnesium Trisilicate	56	
9	Agents acting on the Renin‐Angiotensin system – C09	1438	1.3
	Enalapril	612	
	Lisinopril	336	
	Losartan	335	
	Valsartan	43	
	Captopril	41	
10	Cough and Cold preparations – R05	1160	1.0
	Dextromethorphan	312	
	Acetylcysteine	242	
	Bromhexine	226	
	Codeine, Ephedrine, Promethazine	125	
	Codeine, Promethazine	88	

Only 10.8% of reports specified the patient's outcome, with 2040 (2.0%) and 9159 reports (8.8%) indicating that the patient has yet to recover at the time of reporting, or have recovered, respectively. Out of the 92 (0.1%) reports with fatal outcome, nine were assessed as unrelated to the drug, while 83 could be related to the drug or adverse reaction. For fatal reports assessed as related, SJS/TEN (n = 53, 63.9%) had the highest number of reports. Allopurinol (n = 23, 27.7%) was the top suspected drug.

Table 4 lists the 10 CADRs‐of‐interest and their top 10 suspected drugs. Antibacterials and NSAIDS were frequently associated with all types of CADRs, with the exception of alopecia.^13^, ^14^ Antiepileptics and allopurinol were commonly implicated in SJS/TEN and DHS.

**Table 4:** Ten CADRs‐of‐interest and their respective top 10 suspected drugs^a^

Type of CADRs	No. of suspected drugs	Top 10 suspected drugs (No. of reports of CADR with drug)
Angioedema	17 166	Diclofenac (2159); Paracetamol (1994); Ibuprofen (1797); Naproxen (1555); Aspirin (1474); Mefenamic Acid (839); Cotrimoxazole (584); Amoxicillin (577); Coamoxiclav (420); Ketorolac (322)
Urticaria	8406	Iohexol (652); Coamoxiclav (484); Diclofenac (408); Paracetamol (391); Amoxicillin (352); Aspirin (286); Ibuprofen (259); Ceftriaxone (257); Ciprofloxacin (250); Cotrimoxazole (237)
SJS/TEN	1114	Carbamazepine (126); Cotrimoxazole (84); Allopurinol (80); Phenytoin (53); Omeprazole (46); Coamoxiclav (44); Amoxicillin (41); Etoricoxib (26); Ceftriaxone (24); Lamotrigine (23)
FDE	869	Etoricoxib (95); Cotrimoxazole (94); Paracetamol (67); Doxycycline (47); Coamoxiclav (43); Amoxicillin (29); Tetracycline (27); Ciprofloxacin (23); Mefenamic Acid (19); Diclofenac (15)
AGEP and Pustular Rash	607	Coamoxiclav (69); Amoxicillin (24); Ceftriaxone (24); Cotrimoxazole (24); Clarithromycin (22); Ciprofloxacin (20); Diclofenac (18); Paracetamol (16); Benzylpenicillin/Penicillin G (15); Ibuprofen (15)
Bullous Eruption	562	Cotrimoxazole (56); amoxicillin (26); Tetracycline (26); Coamoxiclav (25); Etoricoxib (24); Paracetamol (18); Diclofenac (17); Doxycycline (16); Aspirin (14); Mefenamic Acid (14)
DHS	325	Allopurinol (57); Phenytoin (39); Cotrimoxazole (33); Carbamazepine (20); Dapsone (14); Sulfasalazine (9); Diclofenac (9); Omeprazole (8); Piperacilin & Tazobactam (7); Vancomycin (6); Coamoxiclav (6), Isoniazid (6); Rifampicin (6)
Photosensitivity	123	Hydrochlorothiazide (37); Doxycycline (10); Fenofibrate (7); Simvastatin (5); Ciprofloxacin (4); Griseofulvin (4); Tetracycline (3); Nifedipine (2); Entecavir (2); Glipizide (2); Hydroxychloroquine (2); Coamoxiclav (2); Amiodarone (2); Atenolol (2); Chlorpromazine (2); Ofloxacin (2)
Alopecia	75	Azathioprine (6); Losartan (5); Leflunomide (5); Amlodipine (4); Atenolol (4); Fluconazole (4); Simvastatin (4); Valproate (3); Nilotinib (3); Acarbose (2); Carbimazole (2); Metformin (2); Imatinib (2); Lisinopril (2); Tolbutamide (2)
Skin Discoloration	62	Simvastatin (5); Cotrimoxazole (5); Amiodarone (2); Laropiprant and Niacin(2); Tetracycline (2); Enalapril (2); Aspirin (2); Ciprofloxacin (2)

a The number of drugs involved ≠ number of reports for CADR as more than 1 drug could be suspected in a single report.

For each CADR‐of‐interest, a subgroup analysis was performed based on age, sex, race, and latency of reaction (Table 5). Alopecia had the lowest male/female ratio at 0.28 while DHS had the highest at 1.40. Angioedema was reported by younger patients (median age = 34 years) while photosensitivity, DHS and SJS/TEN were reported by older patients (median age = 50‐58 years). In general, the racial distribution across each CADR‐of‐interest was consistent with that of Singapore's population, with trending towards the Malays, Chinese and Indians for SJS/TEN, photosensitivity and skin discoloration, respectively. These trends remained even after taking into account the population size^15^ of each race to estimate the frequency of each CADR‐of‐interest across races. The latency period for CADRs also varies, from acute ones (eg angioedema, urticaria) to those which take weeks to occur (eg photosensitivity, alopecia).

**Table 5:** Ten CADRs‐of‐interest and their breakdown by demographic characteristics and latency

Type of CADR	Median age (years)	Sex Ratio (M:F)	Race (%)				Median Latency (days)
			Chinese	Malay	Indian	Others	
Angioedema	34 (0‐103)	0.82	71.5	13.8	8.1	6.6	0.0
Urticaria	41 (1‐101)	0.77	72.3	12.3	7.2	8.2	0.0
SJS/TEN	50 (1‐115)	0.89	67.9	19.9	4.9	7.3	12.0
FDE	45.5 (1‐93)	1.30	76.1	9.3	9.4	5.2	1.0
AGEP and Pustular Rash	48.5 (1‐103)	0.85	67.0	17.0	11.1	5.0	3.0
Bullous Eruption	48.5 (1‐96)	1.20	70.9	15.8	7.7	5.6	2.0
DHS	53 (4‐89)	1.40	72.9	12.1	7.0	8.0	26.0
Photosensitivity	58 (4‐95)	0.65	78.1	12.4	5.7	3.8	31.0
Alopecia	49.5 (11‐81)	0.28	75.0	8.9	10.7	5.4	69.5
Skin Discoloration	47 (1‐89)	0.46	66.0	8.0	20.0	6.0	4.0
Total	41 (0‐115)	0.78	72.5	12.5	8.1	7.0	0.0

Table 5 lists the CADRs‐of‐interest from most to least commonly reported. The median age, sex ratio (M:F), racial distribution, and median latency of each CADR is listed.

CADRs with sex ratio (M:F) greater than one indicate that the CADR is reported more in males than in females, ie FDE, bullous eruption, and DHS.

The racial distribution across each CADR was generally consistent with that of the local population [Chinese (74.1%), Malays (13.4%), Indians (9.2%), Others (3.3%)^16^], with slight deviations noted for SJS/TEN (more frequently reported in Malays), photosensitivity (more frequently reported in Chinese) and skin discoloration (more frequently reported in Indians).

## 4 DISCUSSION

In this study, we analyzed a large volume of spontaneous AE reports and highlighted the various patterns of CADRs and their implicated drugs. Compared to similar studies with smaller sample sizes^8^, ^11^, ^16^ our larger sample size allows for greater power and better capture of the variety of CADRs, specifically the less frequently reported ones.

CADRs are the most commonly reported AEs by organ class, constituting about 60% of all AE reports received by HSA. This is due to CADRs being more visible and easily recognizable, leading to less underreporting as compared to other AEs. The most frequently reported CADR was rashes (inclusive of nonspecified rash, follicular rash, maculopapular rash, vesicular rash) (67.2%), although a large proportion of these were simply reported as “rash”. Typically, such rashes are considered nonserious, and hence reported with scanty information. We also received a disproportionately higher number of AE reports for SCARs compared to other CADRs, suggesting that SCAR cases tend to be reported more conscientiously by healthcare professionals, as it is critical to document these in the electronic medical records to prevent reexposure which could be life‐threatening.

The top implicated drug classes in our study, namely antimicrobials (43.5%), NSAIDs (16.2%) and analgesics (9.0%) are similar to that reported in an Italian study.^8^ These tend to be associated with nonserious CADRs such as angioedema, urticaria, FDE, and bullous eruption (Table 4). In comparison, a study by Ding et al^9^ focusing on CADRs in a tertiary hospital reported antibiotics, antiepileptics and antigout drugs as the top drug classes implicated, highlighting the difference in the propensity of different drug classes to cause serious CADRs requiring hospitalization.

### 4.1 Selected CADRs‐of‐interest

#### 4.1.1 Angioedema

Consistent with literature, our results showed that angioedema was commonly caused by NSAIDs, penicillins, and sulfa drugs.^17^, ^18^ While antibiotics‐induced angioedema is likely due to type I hypersensitivity, NSAIDs‐induced angioedema is considered a nonallergic reaction, attributed to cyclooxygenase (COX) inhibition, leading to the shunting of arachidonic acid toward the production of excessive leukotrienes which are mediators for swelling.^17^, ^18^ This pathophysiology explains why COX‐2 inhibitors are better tolerated compared to nonselective NSAIDs, and our study's observation is consistent with this: Angioedema is commonly reported with nonselective NSAIDs (eg diclofenac, ketorolac, etc.), but not so with COX‐2 inhibitors (eg etoricoxib) (Table 4). As for paracetamol‐induced angioedema, the mechanism is still not well understood, with both IgE‐mediated pathway and leukotriene production being possible.^19^

#### 4.1.2 Urticaria

While angioedema is characterized by deep swelling in the submucosal or subcutaneous tissue, urticaria is associated with transient swelling of the skin.^20^ Drug‐induced urticaria can be immunologically mediated (eg with antibiotics), or nonimmunologically mediated (eg with NSAIDs)^5^, ^21^ Our study also observed radiocontrast media, specifically iohexol, as a frequent causative agent for urticaria. It is postulated that radiocontrast media acts via a nonimmunologic mechanism by triggering direct mast cell degranulation, resulting in histamine release.^20^, ^21^ As such, most cases of symptomatic urticaria can be managed with an antihistamine such as diphenhydramine.^22^

#### 4.1.3 Stevens‐Johnson syndrome, toxic epidermal necrolysis (SJS/TEN) and drug hypersensitivity syndrome (DHS)

While SJS/TEN has multiple causes, most are drug‐induced,^23^ and our study identified antiepileptics (ie carbamazepine, phenytoin, lamotrigine), antibiotics (cotrimoxazole, amoxicillin‐clavulanic acid, amoxicillin, ceftriaxone) and allopurinol as commonly implicated drugs (Table 4). A similar drug profile was also seen in our DHS reports, with sulfa‐drugs (ie cotrimoxazole, dapsone, sulfasalazine) featuring more strongly for DHS. SJS/TEN appear to be more common in females, while the converse is true to DHS. Comparing latency between SJS/TEN and DHS of the top four drugs, latency tends to be longer in DHS than SJS/TEN (median 23 vs 20 days for phenytoin, 28 vs 20 days for allopurinol, 26 vs 7 days for cotrimoxazole and 19 vs 13 days for carbamazepine.)

Human Leukocyte Antigen (HLA) genes have been shown to be associated with drug‐induced SCARs, including SJS/TEN due to carbamazepine, phenytoin, and allopurinol.^23^ The HLA‐B*1502 allele, a well‐known marker for carbamazepine (CBZ)‐induced SJS/TEN, is present in many Asian populations,^24^, ^25^ including Singapore.^26^ The frequency for this allele in Singaporeans is approximately 1 in 5 Malays, 1 in 8 Chinese and 1 in 25 Indians, compared to 1 in 500 Japanese or less than 1 in 1000 Caucasians.^27^ The higher allele frequency in Malays may partly explain the disproportionate number of SJS/TEN cases received: A quarter of the CBZ cases in our study were in Malays, although they comprise only 13.4% of the general population.^16^

Between 2003 and 2012, HSA received an average of 15 reports of CBZ‐induced SJS/TEN per year. Based on strong data from local and international studies supporting the association between the HLA‐B*1502 allele and CBZ‐induced SJS/TEN, the Ministry of Health, in a joint Dear Healthcare Professional Letter with HSA issued in April 2013, stated that genotyping of HLA‐B*1502 prior to the initiation of CBZ therapy in new patients of Asian ancestry is standard of care.^28^ This has mitigated the risk of CBZ‐induced SJS/TEN locally,^29^ illustrating the role a regulatory authority can take in advancing the use of pharmacogenetics for drug safety.

For allopurinol, there is a strong genetic association between HLA‐B*5801 and allopurinol‐induced SCARs. The frequency of this allele in Singaporeans is approximately 1 in 5 Chinese, 1 in 15 Malays and 1 in 25 Indians.^30^ The higher HLA‐B*5801 allele frequency in the Chinese population could partly explain the disproportionately higher number of allopurinol‐SJS/TEN cases seen in our study: 85.5% of these occurred in Chinese patients, although the Chinese make up 74.1% of the general population.^16^ In contrast to CBZ, a cost effectiveness study found that genotyping all gout patients for the HLA‐B*5801 allele prior to initiation of allopurinol is currently not cost‐effective for Singapore's overall population from a health systems perspective. The relatively low positive predictive value of the test and limited alternative urate‐lowering therapies were contributory factors to this.^30^

In the EuroSCAR study, oxicam‐NSAIDs (eg piroxicam) were found to be strongly associated with SJS/TEN.^29^ Conversly, COX‐2 inhibitors, celecoxib and rofecoxib, did not show such an association,^34^ although cases of etoricoxib‐induced TEN have been reported.^32^, ^33^ In our setting, systemic oxicam‐NSAIDs are not widely used, and we found COX‐2 inhibitor etoricoxib as the most frequently reported NSAID associated with SJS/TEN instead.

Sulphonamide antibiotics (eg cotrimoxazole) are well known to cause SJS/TEN, but for amoxicillin and amoxicillin‐clavulanic acid, this is less clear. Half of the SJS/TEN reports we received for the drug class penicillins had co‐suspected drugs, and in some cases, the antibiotic was started only after the prodromal symptoms of SJS/TEN appeared.

Omeprazole was found to be a co‐suspected drug in 60.9% of the omeprazole‐SJS/TEN cases, suggesting it could be an innocent bystander. Similarly, in other studies,^31^, ^34^ it was found that pantoprazole was commonly taken with drugs which carry higher risks for SJS/TEN, and was often not temporally convincing. Other proton pump inhibitors (PPIs) have also been reported to carry nonsignificant risk for SJS/TEN.^31^

#### 4.1.4 Fixed drug eruption (FDE)

Drugs, specifically NSAIDs and antibiotics, are the most common cause of FDEs, and our findings reflect this. Interestingly, a retrospective chart review by the Singapore National Skin Center^35^ also identified etoricoxib as the most common cause of FDE, accounting for 38.7% of 62 FDE patients. In that study, three‐quarters of the patients reacting to etoricoxib were Chinese patients, and this was also observed in our study. The possibility of genetic predisposition was considered, suggesting a genetic association resulting in higher incidence of etoricoxib‐induced FDE in the local Chinese population.^35^

#### 4.1.5 Acute generalized exanthematous pustulosis (AGEP) and pustular rash

In a EuroSCAR study,^36^ aminopenicillins, sulphonamides, and quinolones were found to be highly associated with AGEP, but not paracetamol and cephalosporins. In comparison, our results showed that all these drugs were frequently reported to cause AGEP, although in the paracetamol and ceftriaxone cases, one‐third were accompanied with co‐suspect drugs. While our database did not feature reports of (hydroxy)chloroquine, terbinafine and diltiazem‐associated AGEP prominently (≤5 reports for each drug), the EuroSCAR study did detect them as culprit drugs which have a high risk of causing AGEP. Different prescribing patterns and under‐reporting of AEs could explain this. For similar reasons, we also identified different NSAIDs as causative agents: The EuroSCAR study reported oxicam‐NSAIDs, as opposed to diclofenac and ibuprofen seen in our study.

#### 4.1.6 Bullous eruptions

Bullous eruptions encompass a range of clinical presentations such as pemphigus, bullous pemphigoid, and linear IgA bullous dermatosis.^5^, ^37^ Pemphigus can be triggered via a biochemical pathway by thiol drugs (eg d‐penicillamine, captopril, lisinopril) or phenol drugs (eg rifampicin, aspirin, levodopa), or via an immune‐mediated pathway by non‐thiol drugs (eg cephalosporin, penicillin, enalapril), with both pathways leading to acantholysis.^37^ Bullous pemphigoid, on the other hand, is most often associated with thiol drugs.^5^, ^37^ In linear IgA bullous dermatosis, vancomycin is the most common culprit drug.^37^ While most of our reports of bullous eruptions did not specify the type of bullous disorder, we did receive reports of vancomycin‐associated linear IgA bullous dermatosis, bullous pemphigoid secondary to enalapril, pemphigus vulgaris associated with captopril, as well as with pemphigus with rifampicin.

#### 4.1.7 Photosensitivity

Drugs often associated with photosensitivity (eg tetracyclines, thiazides, chlorpromazine, amiodarone) were also elucidated by our study.^5^, ^38^, ^39^ Although entecavir is not known to cause photosensitivity reactions, there were two such reports in our database, one of which was confirmed with skin biopsy showing deep perivascular dermatitis with eosinophils. The elderly patient was on entecavir for 5 months, and recovered 3 months after cessation of drug. Our results also suggest that patients who are female, Chinese or of older age are more likely to develop and report photosensitivity reactions. The protection offered by melanin in darker‐skinned patients could explain the higher reporting of photosensitivity reactions by the Chinese.^40^

#### 4.1.8 Alopecia

Drug‐induced alopecia could be categorized into anagen effluvium (ie hair growth phase) or telogen effluvium (ie resting phase).^41^ Anagen hair loss is often dose related and commonly associated with chemotherapy, but reports of chemotherapy‐induced alopecia are lacking from the AE database. One reason is that expected reactions such as these tend to be under‐reported. Telogen hair loss is linked to a wider variety of endogenous and exogenous factors, such as major surgery, serious illness, childbirth, malnutrition, stress, and drugs (eg beta blockers, valproic acid, leflunomide), hence it is more difficult to pinpoint a drug as the causative agent.

Of the six azathioprine reports, four of them were accompanied by pancytopenia or severe neutropenia. Interestingly, in a study on azathioprine side effects in Chinese patients with systemic lupus erythematosus, five out of 126 patients exhibited alopecia, and all five developed leukopenia subsequently. Although it cannot be conclusively said that alopecia and leukopenia are related due to limited data, the study suggested that alopecia could be a sign of hematological toxicity, prompting early reduction or cessation of azathioprine.^42^

#### 4.1.9 Skin discoloration

Drugs often implicated in causing skin discoloration are amiodarone, tetracycline, NSAIDs, antimalarials, and psychotropic drugs. Amiodarone is associated with slate‐gray pigmentation in unprotected light exposed skin areas, especially in light‐skinned individuals on prolonged therapy. This hyperpigmentation is due to the accumulation of amiodarone and its metabolites in the skin.^43^, ^44^ Long term use of tetracyclines are also known to cause abnormal pigmentation. With minocycline, especially when used long term for treatment of acne, pigments are deposited as a result of an insoluble minocycline‐melanin complex.^45^ Our study found that females are more likely to report drug‐induced skin discoloration. Although Indians make up about 9.2% of the local population,^16^ there appears to be a greater proportion of reports of skin discoloration from Indians (20.0%).

## 5 CONCLUSION

While our analysis did not detect any new associations or drug signals, it is interesting to note that CADRs such as SJS/TEN, photosensitivity and skin discoloration seem to be reported more frequently in Malays, Chinese, and Indians, respectively. However, given the limitations of the spontaneous reporting system, these observations should be taken into context. To overcome these limitations, HSA is looking into leveraging on electronic medical records to detect ADRs, including SCARs. With information on race, diagnoses, and medication history, it will become possible to explore the distribution of various CADRs across different races.

In summary, we have analyzed a large dataset of over 100 000 CADR reports from a 10‐year period and identified the types of CADRs reported, as well as their associated drugs, latency periods, and patient characteristics. Such information could add value to healthcare professionals as they assess CADR cases and evaluate suspected drugs. Timely and accurate identification of the causative drug can enable HCPs to take the appropriate actions and improve patient clinical outcome.

## DISCLOSURE

None declared.
